# #Antivaccination on Instagram: A Computational Analysis of Hashtag Activism through Photos and Public Responses

**DOI:** 10.3390/ijerph17207550

**Published:** 2020-10-17

**Authors:** Yunhwan Kim, Donghwi Song, Yeon Ju Lee

**Affiliations:** 1 Division of Media Communication, Hankuk University of Foreign Studies, Seoul 02450, Korea; yunhwankim2@hufs.ac.kr; 2 Business School, Hankuk University of Foreign Studies, Seoul 02450, Korea; 201501686@hufs.ac.kr; 3 Division of Applied Mathematical Sciences, Korea University, Seoul 30019, Korea

**Keywords:** antivaccination, Instagram, hashtag activism, Microsoft Azure Cognitive Services, engagement, comment sentiment

## Abstract

A dramatic increase has been registered in the number of social media posts in photo form as well as in hashtag activism. Hashtags, which manifest thoughts and feelings clearly and concisely, originated on Twitter, where the length of a post is limited; their use, however, has expanded into other social media services, including Instagram. Hashtags, which make it easy to find and express support for posts of interest, have been widely used for online activism, although they have been criticized for fostering confirmation bias. Moreover, hashtag activism in photo form has been relatively understudied. This research analyzed Instagram photos with antivaccination hashtags as an example of hashtag activism through photos. In addition, we examined how the photo features were related to public response, which was manifested via engagement and comment sentiment. The results suggest that the photos which were categorized into “text” took the largest share. It was also found that the major way of claiming was to imprint key messages that persuade people not to vaccinate with remarks from professionals on photos and provide a source of supporting information in the post text with hashtags of antivaccine intention. Various photo features showed associations with engagement and comment sentiment, but the directions of correlation were usually the opposite: these results suggest that engagement and comment sentiment are separate domains that reveal different public responses.

## 1. Introduction

Social media has been playing an increasingly influential role in expressing one’s opinion online, and the means of expression utilizing the functions of social media are various, with hashtags being one of the most popular ones. A hashtag is a brief keyword prefixed by the symbol # (hash) included in social media posts in order for them to be easily searched for among the huge volume of posts on the platform [1]. The hashtag is known to have been first introduced by Twitter, although it is now widely used on other social media services. It serves not only as a guide for finding and following posts of interest but also as a marker that shows one’s stance on an issue explicitly and succinctly [1]. These characteristics make the hashtag a useful tool for activists who need an efficient way to widely disseminate their belief and interactively communicate with the public.

Hashtag activism is a realization of this potential—it is an attempt to use the hashtag functions in social media to incite social change [2]. Individual users or activists devise tags that manifest their belief on an issue concisely and attach them to their social media posts; similarly, users or activists who support or approve of their belief use the same tag to show their endorsement. Like other memes on social media [3], some of these tags are shared by the general public and spread throughout the social media space, and some of them, including #MeToo or #BlackLivesMatter, have gone viral beyond online spaces, becoming symbols of social movements. The hashtag has been acknowledged as one of the major forms of online activism [4].

However, hashtag activism has also been criticized. It has been argued that the effectiveness of hashtag activism is questionable [5], and that activism by short hashtags may obscure the rich context of a movement [2]. Yet, one of the biggest criticisms is that it can foster confirmation bias [6]. Hashtags make it easy to find posts of interest, but this function may worsen the tendency of networking only with likeminded users or paying attention only to posts with similar viewpoints. This can result in polarization [7,8], which may harm society at large.

The antivaccination movement can be considered an example of this phenomenon. Activists upload posts with hashtags displaying antivaccine intentions on social media, and these are used by other social media users who share their belief. Such activism via hashtags can foster confirmation bias, and concern has been increasing about its possible harm to the society—for example, the breakdown of herd immunity. In this regard, the interest in the antivaccination movement on social media has been considerable, and many studies have analyzed the social media data of the movement [9,10,11].

It must be noted that most of the previous studies about hashtag activism in general and antivaccination on social media in particular have analyzed text data. While text is still the major form of online data, it is also true that the share of visual data such as photographs has been rapidly increasing, and photo-centric social media services (e.g., Instagram) have attracted many users, significantly growing their impact. Yet, hashtag activism via photos on social media has been relatively understudied. Furthermore, while the literature has paid much attention to the role of social media photos in public health [12], the vast majority of previous studies about photo content have used the content analysis method with human coders, whereas computational analysis has been employed in a limited number of studies [13,14]. Meanwhile, the relationship between features of the photos and the engagement of the public has been understudied. Public engagement, which is usually measured by the number of likes and comments on social media [15], can show what kind of photos would induce public endorsement through photos of hashtag activism; however, little research has been conducted on it, especially in the literature on the antivaccination movement.

In this regard, the present study aims to explore how the antivaccination movement is represented on Instagram. In addition, it aims to investigate the relationship between the features of antivaccination Instagram photos and public engagement. With these aims, Instagram photos with antivaccination hashtags were downloaded, and photo features at various levels were extracted. Moreover, the current study measured the public engagement with the photos as well as the sentiment of the comments on the photos; finally, it analyzed how photo features are associated with them, and whether these features can predict them. The following research questions are proposed:

RQ1. What are the key characteristics of the content of Instagram photos with antivaccination hashtags?

RQ2. What are the relationships between the features of Instagram photos with antivaccination hashtags and the engagement of the public with the photos?

RQ3. What are the relationships between the features of Instagram photos with antivaccination hashtags and the sentiments of comments on the photos?

This study is structured as follows. First, it reviews previous studies about social media photos and public health, hashtag activism, and the antivaccination movement on social media. Next, it describes how the data were gathered and which features were extracted and used for the analysis of the Instagram photos. Furthermore, it presents the key characteristics of the content of Instagram photos, the associations of photo features with public responses, and the predictability of photo features regarding engagement and comment sentiment. Finally, it discusses the implications of the results.

## 2. Related Works

### 2.1. Social Media Photos and Public Health

Many previous studies have paid attention to social media photos of topics pertaining to public health. First, photos about diseases were analyzed. Yi-Frazier et al. [16] asked 20 adolescents with type 1 diabetes to post diabetes-related photos for three weeks on Instagram. They analyzed the uploaded photos and found that their major categories were diabetes care, humor, and food. Seltzer et al. [17] analyzed the Instagram photos with the #zika tag and found that they were primarily related to the prevention and transmission of the disease. Fung et al. [18] also analyzed the content of Zika-related photos and found that its prevention, its effects on pregnancy, and the deaths associated with it were major categories. Instagram posts with the #melanomasucks tag were analyzed by Cho et al. [19], with the most frequent content being controlling melanoma and the effects of having melanoma. Nobles et al. [14] examined the demographic profile of faces in Instagram posts with the #HIV tag and compared it with the profile of those at most risk of a new HIV diagnosis. Their results suggest that the faces on Instagram photos were older, more female, more white, less black, and less Hispanic than those of new HIV diagnoses.

Photos about cigarettes have also been analyzed in previous research. Chu et al. [20] categorized Instagram photos about the electronic cigarette and found that advertisement was the most common type of content, followed by product and activity. The results of Lee et al. [21] also revealed that the most popular category of Instagram posts of electronic cigarettes was marketing. Laestadius et al. [22] examined what kind of user mainly posted about electronic cigarettes on Instagram; it was found that over half of the posts were uploaded by corporate users. Allem et al. [23] analyzed the content of Instagram photos of cigars and found that about one fourth of the photos were about marijuana.

Next, previous studies have analyzed social media photos related to the human body. For example, they examined photos uploaded on Instagram by body-positive accounts [24] and health influencers [25] and investigated what kind of body images these accounts convey. Self-injury is another topic that previous studies on Instagram photos have focused on, showing that pictures with a high degree of injury generated more comments [26], though the majority did not contain any advisory warning [27]. Previous studies also analyzed social media photos about alcohol [28], junk food [29], pregnancy [30], and surgery [31].

It must be noted that, however, most of those studies used the content analysis method with human coders, whereas advanced computational analysis methods were used in a limited number of studies. To name a few, Kim and Kim [13] analyzed the photos on the CDC (Centers for Disease Control and Prevention) Instagram account using various features at the content and pixel levels which were extracted by computational methods. Moreover, Nobles et al. [14] analyzed the faces in Instagram photos with the #HIV hashtag with automated methods and compared the demographics of the faces with the ones at risk of diagnosis. The current study is in line with the abovementioned research, adopting computational methods to analyze photos about antivaccination.

### 2.2. Hashtag Activism

Since hashtags have been widely used in a variety of movements, many previous studies have examined hashtag activism using diverse methods. A group of studies have used “hashtag ethnography” [32]—an ethnographic description of how hashtags have been used in social movements. They have investigated movements mainly concerned with race [32,33] and gender [34,35,36], as well as movements by students [37] and teachers [38].

Another group of studies analyzed the content of hashtagged social media posts. Papacharissi and Oliveira [39] examined the form of news exhibited under the hashtag #egypt on Twitter. Giglietto and Lee [40] analyzed tweets containing the #JeNeSuisPasCharlie hashtag and revealed the various discursive strategies that the users of the hashtag employed. Brown et al. [41] conducted a content analysis of tweets with the hashtag #SayHerName, focusing on the Black women who are victims of police violence. Xiong et al. [42] analyzed tweets with the #MeToo hashtag and identified its themes, which included the antecedents of the main event and suggested actions for women’s rights. Ince et al. [43] analyzed the content of #BlackLivesMatter tweets and categorized them into five groups, including solidarity or approval of the movement. Ray et al. [44] also examined #BlackLivesMatter tweets and showed the evolution of both anti- and pro-police narratives on Twitter. This coexistence of counter- and counter-counter-narratives is also visible in the work of Drüeke and Zobl [45], who analyzed tweets with #aufschrei, which means “cry out”; the hashtag was originally used to encourage critical voices against sexual assault, but a persistent number of anti-feminist and sexist messages also employed the hashtag.

A network analysis method has also been used in the literature about hashtag activism. Concerning the #wirecall hashtag, which was used in activism around the 2012 Wisconsin recall election, Xu et al. [46] built and analyzed the follower-following network on Twitter. Their results showed that users with a higher level of connectivity were more involved in the information flow. Jackson and Welles [47] analyzed the network of retweets and mentions with the #Ferguson hashtag on Twitter and found that the network was a broadcast network, where a small number of influential users transmit messages to the vast majority of users unilaterally. Yan et al. [48] also analyzed the network of retweets and mentions on Twitter to reach similar results, revealing that the #ConcernedStudent1950 hashtag network has evolved to be highly concentrated. Wang et al. [49] paid attention to the hashtag co-occurrence network; their analysis of tweets with #OccupyWallStreet revealed that the role of hashtags in information virality may vary with the context of the tweets. Further, concerning the #OccupyWallStreet hashtag, Tremayne [50] generated a bimodal network whose nodes are one of the two kinds—user account or hashtag—and identified a small number of key nodes that are important for the spread of the movement.

While hashtag activism has been examined in previous research, studies that have paid attention to hashtag activism through photos are still rare. The work by Stefanone et al. [51] is one of the exceptions; they analyzed the photos with the #guncontrol hashtag on Twitter and showed that photos with an attribute frame, fear and humor appeals, and positive valence were retweeted more. Moreover, Ichau et al. [52] analyzed the content of Instagram photos with the #jews hashtag, finding that the photos can be categorized into five groups, including people and private lives, culture and history, and cultural production. The present research is in line with these studies and investigates hashtag activism through photos using computational methods.

### 2.3. Social Media Data Analysis on Antivaccination Movement

Social media has a major impact on the way that people interact and exchange information, and this is also the case in the antivaccination debate [53]. Since information on the internet may influence people’s behavior regarding vaccination, it is important that facts are shared; yet, the social media platforms that enable individual users to find and share information easily can have two side effects on vaccination decision: they can lead people to get vaccinated [54], but antivaccination movements also can be strengthened [55,56]. Thus, it is relevant to observe the vaccination debate on social media and to examine how antivaccination movements proceed [57].

In this regard, previous studies have analyzed the social media posts about the antivaccination movement. Keelan et al. [58] analyzed the Human Papilloma Virus (HPV) vaccination debate on Myspace blogs and found the difference in the points that positive and negative blogs made; positive blogs focused on the effectiveness of the vaccine, whereas negative blogs focused on its risks. Salathe and Khandelwal [9] conducted a sentiment analysis of tweets about H1N1 vaccination and found that users who posted about vaccination generally formed communities, and that most communities were dominated by either positive or negative sentiments. Radzikowski et al. [10] presented the Twitter narrative regarding vaccination after the 2015 measles outbreak; the major themes included the political aspects of the vaccination, anti-antivaccination activism, and more health-oriented issues. Broniatowski et al. [59] examined how Twitter bots promote online vaccination debate and found that bots and “content polluters” tweeted more about vaccination than average users. Jang et al. [60] compared the vaccine-related content in Twitter, Reddit, and online news in the US, Canada, and the UK, and showed that both Twitter and Reddit discussed the vaccine–autism link more than online news.

A network analysis method has been used to observe antivaccination movements on social media. Yuan et al. [61] analyzed the retweet network related to the MMR vaccine; they discovered that users with pro- and antivaccine views retweet much more from their own opinion communities. Himelboim et al. [62] also found subgroups regarding the HPV vaccine conversation on Twitter, noting that interactions and information flows mainly occurred inside each subgroup. Johnson et al. [63] revealed the difference in the network clusters; antivaccination clusters were smaller in size but highly entangled with undecided clusters, whereas pro-vaccination clusters were more peripheral. Francia et al. [64] showed that opinion groups regarding vaccination could be characterized according to their political perspectives. Smith and Graham [65] examined the discourses present within anti-vaccination Facebook pages using social network analysis and found that the present-day discourses were located at the center of discourses.

While antivaccination movements on social media have been investigated in previous studies, limited research has focused on social media photos about antivaccination. Guidry et al. [66] analyzed vaccine-related photos on Pinterest and showed that most photos were concerned with antivaccination. Kearney et al. [67] conducted a sentiment analysis of the Instagram photos about the HPV vaccine and showed that the proportion of pro-vaccine posts was higher than that of the antivaccine posts, but the pro-vaccine posts were liked less than the antivaccine posts. Chen and Dredze [68] analyzed vaccination photos on Twitter, showing that the sentiment of a photo could be one of the predictive factors of whether the photo would be retweeted. Following these studies, the present research analyzes antivaccination photos on Instagram, employing features both at the content and pixel levels for analysis and investigating their associations with and the predictability of public responses to the photos.

## 3. Method

### 3.1. Data Collection

We crawled the Instagram posts with antivaccination hashtags; Instagram-scraper (https://github.com/rarcega/instagram-scraper) was used for this purpose. Data were gathered from 13 to 20 June 2019. In order to obtain a pool of hashtags about antivaccination, we examined the tags from the best-hashtag.com website (http://best-hashtags.com/hashtag/antivaccine/). After removing hashtags which are unsuitable or too general from the pool, we manually inserted each hashtag in the Instagram search bar and inspected the number of posts with the hashtag. Then, we selected hashtags with a sufficient number of posts for data gathering. As a result, the following hashtags were used for searching: #antivaccination, #antivaccine, #antivax, #antivaxmemes, #antivaxxer, #informedchoice, #informedconsent, #vaccineinjuryawareness, and #vaxxed. After removing duplicates, 96,302 photos in total were used for analysis, and 513,694 comments and 12,579,345 likes accompanying the photos were also collected and analyzed.

### 3.2. Photo Features

#### 3.2.1. Content Category

For a given photo, its content was categorized into one of the predetermined classes; Computer Vision API in Microsoft Azure Cognitive Services (https://azure.microsoft.com/services/cognitive-services/computer-vision/) [69] was used for this purpose. Each photo was uploaded to the server and the pretrained artificial intelligence service categorized its content into one of following 15 classes: abstract, animal, building, dark, drink, food, indoor, others, outdoor, people, plant, object, sky, text, or transportation.

#### 3.2.2. Face Features

From a given photo, we extracted features regarding human faces using Face API in Microsoft Azure Cognitive Services (https://azure.microsoft.com/services/cognitive-services/face/). The pretrained artificial intelligence service detected human faces from each photo, and features including age, gender, and size were measured. Additionally, the emotions expressed on each face on the photos were determined; the sum of all emotion scores was 1.

The face features used for the analysis were as follows: (1) the *number of faces* was measured by counting how many faces were on a given photo, (2) *closeup* was the ratio of the size of the biggest face to the size of the photo, and (3) *face ratio* was the ratio of the sum of the sizes of all faces to the size of the photo. Then, (4) *age* was the average age of all appearing faces, and (5) *female* was the count of female faces in a given photo. The emotion scores of each emotion classes, (6) *anger*, (7) *contempt*, (8) *disgust*, (9) *fear*, (10) *happiness*, (11) *neutral*, (12) *sadness*, and (13) *surprise*, were averaged across all the detected faces in a given photo.

#### 3.2.3. Optical Character Recognition Features

The English words appearing in a given photo were detected using the Optical Character Recognition (OCR) function of Computer Vision API in Microsoft Azure Cognitive Services. The *number of words* feature was measured as the number of English words detected in a given photo.

#### 3.2.4. Pixel Features

The pixel information was processed to extract low-level features using the Python programming language and OpenCV library [70]. The numbers contained in each pixel of a digital photo represent colors—RGB (red, green, blue), HSV (hue, saturation, value), or others according to the color space model—and various features of a photo can be extracted using these numbers.

First, the means and variances were calculated for the red, green, and blue in each photo’s pixels; thus, the resulting features were (1) *red mean*, (2) *red variance*, (3) *green mean*, (4) *green variance*, (5) *blue mean*, and (6) *blue variance*. In the same way, the means and variances were calculated for the saturation and value (i.e., brightness) in each photo pixels; the resulting features were (7) *saturation mean*, (8) *saturation variance*, (9) *value mean*, and (10) *value variance*. Since hue, unlike saturation and value, is a nominal feature, its total range (0 to 179 in OpenCV) was divided into intervals (7, 23, 35, 90, 136, 169), each of which correspond to each key color: red, orange, yellow, green, blue, and violet [71]. The share of pixels whose hue falls into each color interval was calculated; the resulting features were (11) *red share*, (12) *orange share*, (13) *yellow share*, (14) *green share*, (15) *blue share*, and (16) *violet share*. Further, the share of red, orange, and yellow was calculated to be (17) the *share of warm colors*, and the share of green, blue, and violet was calculated to be (18) the *share of cold colors*. In addition, the number of peaks in a hue histogram [72,73] ((19) *hue peaks*) was also employed for analysis; we generated the hue histogram, smoothed it with Kernel Density Estimation, and counted how many local maximums were in the smoothed hue histogram [71]. Finally, the affection features from the PAD (pleasure, arousal, and dominance) model were calculated using the formula (Pleasure = 0.69 × Value + 0.22 × Saturation; Arousal = −0.31 × Value + 0.60 × Saturation; Dominance = −0.76 × Value + 0.32 × Saturation) from previous research [74]; the features were (20) *pleasure*, (21) *arousal*, and (22) *dominance*.

#### 3.2.5. Visual Features

The current study examined the features that represent the attractiveness of a given photo [75]. First, (1) *brightness* measured how bright a given photo was using the average of luminance (Y values in the YUV color space) in the pixels of the photo. Next, (2) *colorfulness* measured how colorful a given photo was using the relative amounts of red, green, and blue values in the pixels and their means and standard deviations [76]. (3) *Naturalness* measured the degree of correspondence between a given image and the human perception of reality [77]; we counted the proportion of pixels whose saturation and luminance were located in a certain range [75]. (4) *Contrast* and (5) *RGB contrast* were also measured; the former was calculated by the ratio of the standard deviation of the luminance in pixels to the number of pixels [75], and the latter was calculated by extending the former into RGB color space with three dimensions. (6) *Sharpness*, which represents the clarity and how detailed a given photo is, was calculated by getting the Laplacian of each pixel’s luminance and normalized it with the average luminance of neighboring pixels [78].

Concerning color, two additional metrics were also employed for analysis. (7) *Color diversity*, which represents the degree of diversity in the colors of a given photo, was measured by fractal dimension using the box-counting method [79], as has been done in previous studies [71,80]. (8) *Color harmony*, which represents the degree of harmony among the dominant colors in a given photo, was measured by the geometric characteristics that were generated by the dominant color on the color wheel [81]. For this, we generated a hue histogram, smoothed it with Kernel Density Estimation, and identified the highest and the second highest peaks that represent the top two dominant colors. We located the top two colors on the color wheel and measured the internal angle between the two colors as the color harmony [82].

#### 3.2.6. Engagement

The first measure of public response is engagement, which shows the connection between a social media message and the action of the public [83]. As in previous research [15], we measured engagement as the number of likes and comments, which represent online behaviors responsive to a message.

#### 3.2.7. Comment Sentiment

Another measure of public response is the sentiment expressed in the comment on a given post. Sentiment analysis was conducted using Text Analytics API in Microsoft Azure Cognitive Services (https://azure.microsoft.com/services/cognitive-services/text-analytics/). For each comment, it returned a score between 0, which represents being most negative, and 1, which represents being most positive. The sentiment scores across all comments on each photo were averaged and employed for analysis.

## 4. Results

### 4.1. The Content of Antivaccination Instagram Photos

**Figure 1:**
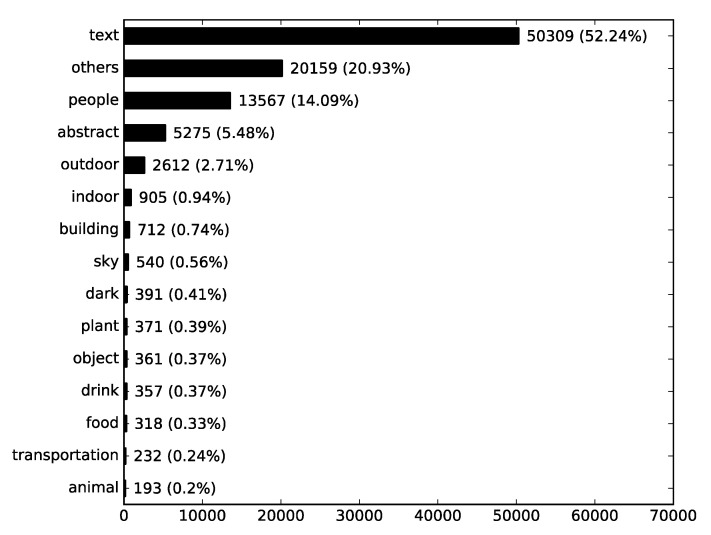
Frequency of antivaccination Instagram photos by content category.

The frequency of the antivaccination Instagram photos by content category is presented in Figure 1. This shows that “text” photos make up the largest part (52.24%), as more than half of the photos had words on them. And, besides “others” category, “people” photos follow (14.09%).

**Figure 2:**
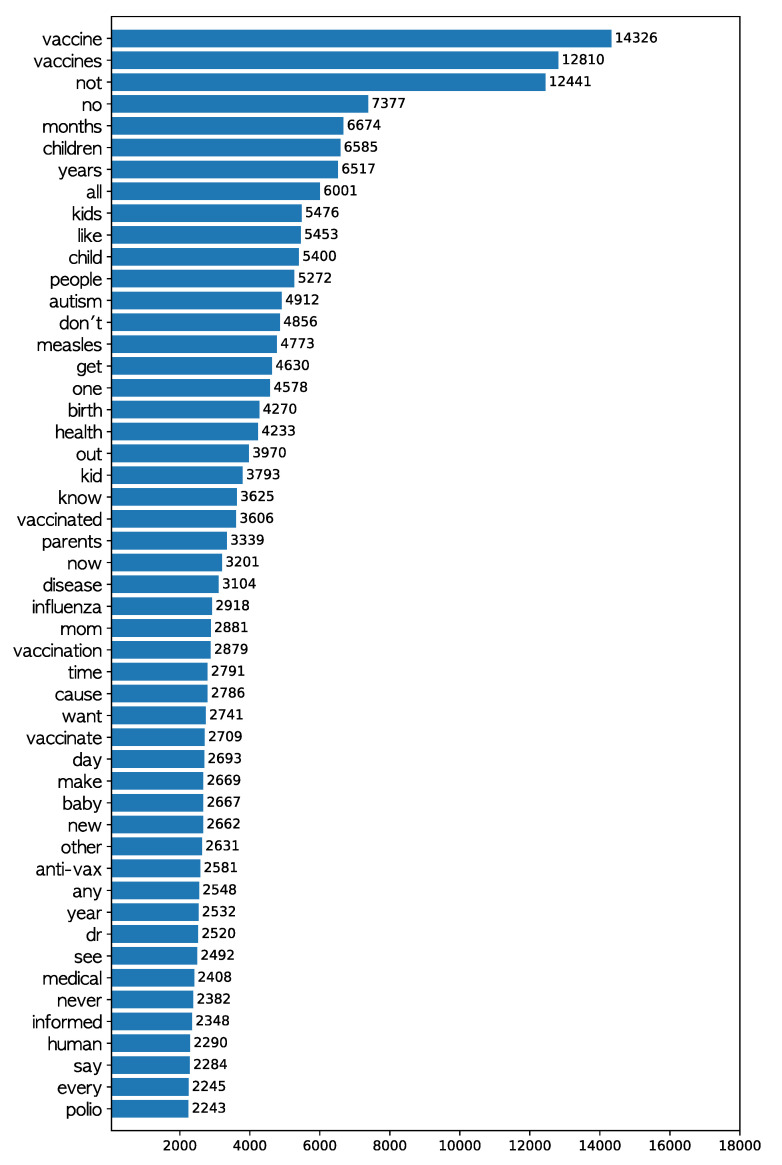
Top 50 frequent words detected by OCR (Optical Character Recognition) from the antivaccination Instagram photos.

Considering that “text” photos make up the largest share, the messages delivered by the words on photos (rather than the post text) were examined. The total number of photos from which OCR detected any word was 78,581, and a total of 2,595,259 detected words were analyzed. Since the OCR detects words one by one without contextual information, the detected words were transformed into lowercase without further preprocessing. Punctuation marks and stop words (&; -; 0; 1; 10; 12; 2; 3; 4; 5; 6; a; about; after; am; an; and; are; as; at; b; be; because; been; but; by; c; can; do; e; et; for; from; had; has; have; he; her; his; how; i; if; in; into; is; it; it’s; i’m; just; may; me; more; my; of; on; only; or; our; s; she; should; so; than; that; the; their; them; there; they; this; to; up; us; v; was; we; were; what; when; which; who; why; will; with; would; you; your; —; •) were removed. Here, a custom list of stop words was used, because the results seemed to contain much noise, which may be due to the errors in OCR. The frequency of the remaining words is presented in Figure 2. The figure shows that many words relate to children: “child”, “children”, “kid”, “kids”, and “baby”. It also demonstrates that “autism” was ranked high in the frequency ranking. Detected words such as “doctor” and “dr” stand for the professionals that might be remarked on concerning the antivaccination movement. Words that prohibit actions—“not”, “no”, “never”, and “don’t”—were also observed, and the key issues highlighted were “measles”, “polio”, “flu”, and others.

**Figure 3:**
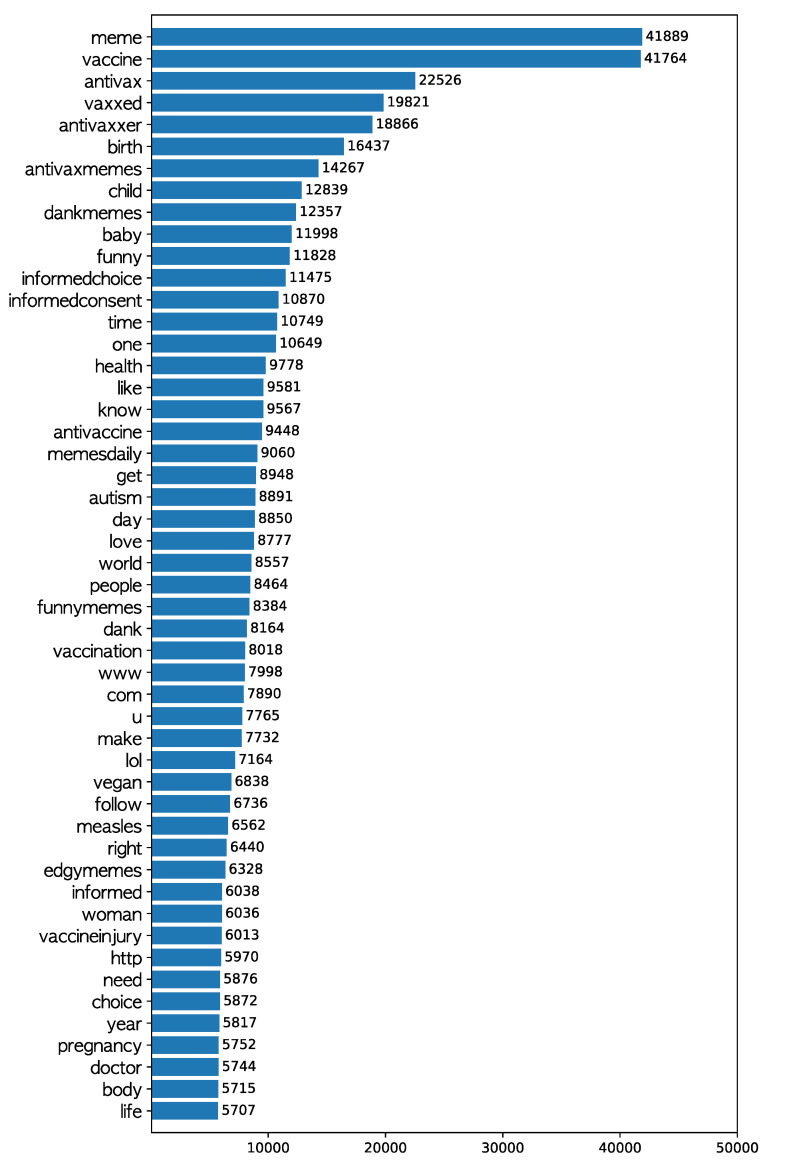
Top 50 frequent words in the text accompanying antivaccination Instagram photos.

We also analyzed the text accompanying to the photos for the purpose of comparison. Each post texts were tokenized into words, transformed into lowercase, lemmatized, and had punctuation marks and English stop words (listed at https://gist.github.com/sebleier/554280) removed. The frequency of the remaining words is presented in Figure 3, suggesting that words (including hashtags) that significantly reveal the antivaccine intention, such as “antivax”, “antivaxxer”, “antivaxmemes”, “informedchoice”, and “informedconsent”, appeared in the post texts. It also shows that the words for Uniform Resource Locations (URLs), such as “http”, “com”, and “www”, appeared frequently, as well as those related to children, such as “birth”, “baby”, and “child”. Taking the results from the analysis of OCR and post texts into consideration together, persuading people not to vaccinate through words imprinted on photos including remarks from professionals and providing the source of supporting information in the post text with hashtags of antivaccine intention seems to be the major means of delivering messages in antivaccination movements on Instagram.

**Figure 4:**
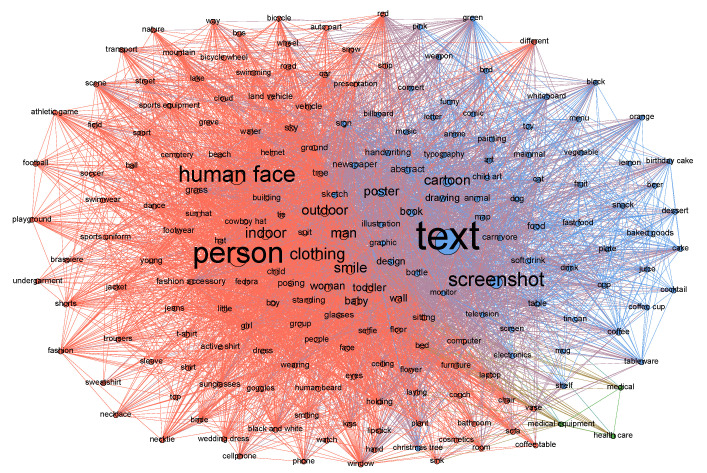
Content tag network of the antivaccination Instagram photos. Node size represents the weighted degree, and nodes with the same color belong to the same subgroup, determined by community detection using the Louvain algorithm. To make it easier to see, only nodes with the top 15% of weighted degree (occupying 95.01% of the total weighted degree) were displayed.

In addition, the content tags network was generated and analyzed. First, for each photo, the tags corresponding to the content of the photo were acquired using Computer Vision API from Microsoft Azure Cognitive Services. Next, only tags whose confidence score was greater than or equal to 0.9, which stands for a high degree of correspondence with the photo content, remained. Networks were generated by connecting pairs of tags using a set of criteria defining co-occurrence—for example, if tag A and tag B were suggested by Computer Vision API with a confidence score of over 0.9, two tags became the nodes of the network and an undirected, weighted link was placed between the two nodes (see Figure 4). Finally, the network was analyzed. A centrality analysis was conducted to determine the central words using four centrality measures: weighted degree centrality, betweenness centrality, closeness centrality, and eigenvector centrality.

The result in Table 1 suggests that the content of the photos was primarily textual. The words that represent the subject in the photos were mostly “person”, “man”, “woman”, “baby”, and “toddler”, whereas the words that represent the action and the location in the photos were mainly “indoor”, “outdoor”, “wall”, “ground”, “grass”, “clothing”, “smile”, and “sitting”. In addition, community detection was conducted using the Louvain algorithm [84] to detect the main themes of the content tag network. The result is presented in Table 2. It suggests that the major themes in the content tag network were text and joy, personal and indoor life, and medical issues.

### 4.2. Photo Features and Engagement of Antivaccination Instagram Photos

**Figure 5:**
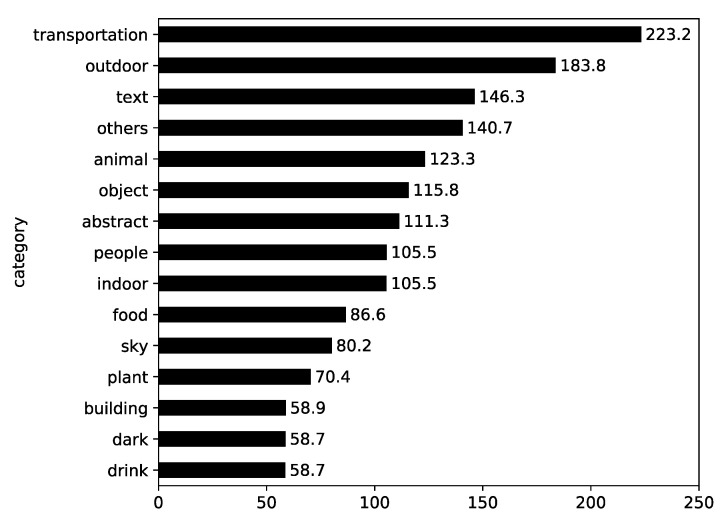
Mean engagement by content category.

First, the mean engagement by content category was investigated. In Figure 5, it is shown that Instagram photos that were categorized into “transportation” and “outdoor” induced the most public engagement, and that photos in the “text” category were also relatively high in terms of mean engagement. The degrees of engagement differed by content category (F = 4.043, *p* < 0.001).

Next, the association between photo features and engagement was examined, and the results are presented in Table 3. From the table, we can observe that most face features were not associated with engagement; yet, the exceptions were age and neutral, as photos that contained faces of older people and those without particular emotion induced more engagement. Moreover, the positive correlation between the number of words and engagement suggests that photos with more imprinted words generate higher engagement. Concerning pixel-level features, the means of red, green, and blue were significantly associated with engagement. We can also observe from the table that photos with splendid color drew less engagement; saturation mean, all color shares (except violet share), and colorfulness were negatively associated with engagement. In contrast, brighter photos induced more engagement, as value mean and brightness were positively correlated with engagement. The affections from the PAD model showed significant associations; pleasure was positively correlated with engagement, whereas arousal and dominance were negatively associated with it.

Finally, predictive models were built and analyzed to investigate how accurately photo features predict engagement. Support vector regression models were trained with 10-fold cross validation, and their root mean square errors (RMSEs) are presented in Table 4. Considering the means and standard deviations of likes (M = 130.624, SD = 891.051), comments (M = 5.334, SD = 22.644), and engagement (M = 135.958, SD = 901.344), we can conclude that the RMSEs are relatively small and that photo features have an acceptable level of predictability in terms of engagement.

### 4.3. Photo Features and Comment Sentiment of Antivaccination Instagram Photos

**Figure 6:**
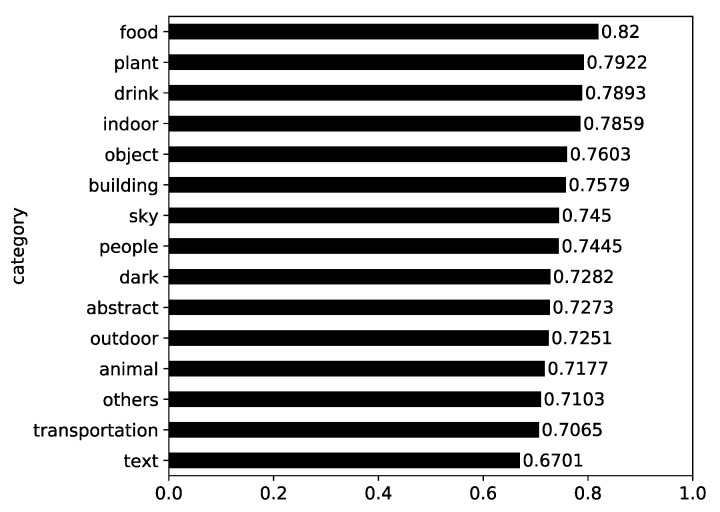
Mean comment sentiment by content category.

First, the mean comment sentiments by content category were investigated. In Figure 6, it is shown that the photos categorized into “food” and “plant” received the most positive comments from the public. Furthermore, we can observe from the figure that the comments on the photos in all categories were positive (above 0.5), although it must be noted that the photos in the “text” category, which were largest in number and ranked high in mean engagement, registered the least positive comment sentiment. The comment sentiments differed by content category (F = 85.546, *p* < 0.001).

Next, we examined the association between photo features and comment sentiment, and the results are displayed in Table 5, which shows that most face features were significantly associated with comment sentiment rather than engagement. The number of faces, age, and female showed positive correlations with the comment sentiment. Moreover, it is observed that happiness was positively associated with it, whereas most negative and neutral emotions—disgust, fear, sadness, surprise, and neutral—were negatively associated with comment sentiment. The negative correlation between the number of words and comment sentiment suggests that photos with more words on them have comments with more negative sentiment; this result is also opposed to the one obtained in the analysis of photo features and engagement. Concerning pixel-level features, the means and variances of red, green, and blue were significantly associated with comment sentiment. In the case of means, the associations were negative, which was the opposite in engagement. From the table, we can also observe that photos with splendid color induced more positive comments from the public, as the saturation mean, all color shares (except the blue share and violet share), and colorfulness were positively associated with comment sentiment. In contrast, brighter photos drew more negative comments, as the value mean and brightness were negatively correlated with the comment sentiment. The affections from the PAD model showed significant associations; pleasure was negatively associated with comment sentiment, and arousal and dominance were positively associated with it. This was also opposite in engagement. The positive correlation of hue peaks suggests that photos that feel mussier had more positive comments, and the visual features showed significant associations with comment sentiment—naturalness and sharpness were positively associated with comment sentiment, and contrast and RGB contrast were negatively associated with it.

Finally, predictive models were built and analyzed to investigate how accurately the photo features predict comment sentiment. Support vector regression models were trained with 10-fold cross validation and their RMSEs are presented in Table 6. Considering the means and standard deviations of the comment sentiment (M = 0.699, SD = 0.251), we can conclude that the RMSEs are relatively large and that the predictability of photo features on comment sentiment is unsatisfactory.

## 5. Discussion

The share of social media posts in photo form has been increasing dramatically, and this is also the case in hashtag activism. Hashtags, which manifest thoughts and feelings clearly and concisely, originated on Twitter, where the length of a message is limited, but their use has been expanded to other social media services, including Instagram. Hashtags, which make it easy to find and express support for posts of interest, have been widely used for online activism, but hashtag activism in photo form has been relatively understudied. This study analyzed Instagram photos with antivaccination hashtags as an example of hashtag activism through photos. In addition, we examined how the photo features were related to public response, which was manifested via engagement and comment sentiment. The major findings and discussions about them are as follows.

First, the photos that were categorized into “text” took the largest share (more than half) among Instagram photos with antivaccination hashtags. This indicates that, while antivaccination posts were uploaded on the photo-centric platform, the photos manifested their idea mainly through the text imprinted on them. One may argue that this is not the best way to exploit the potential of the photographic medium, and that using a photo-centric platform then becomes meaningless. Yet, this can be an efficient way of using the characteristics of visual data, because texts imprinted on photos usually draw attention more easily due to their large size and variety in color and font.

Next, the primary means of delivering messages in antivaccination Instagram posts was found to be imprinting key messages persuading people not to vaccinate through words imprinted on the photos, including remarks from professionals, and providing the source of more information in the post text with hashtags of antivaccine intention. The results revealed that the photos contained words that refer to professionals and that prohibit actions, as well as showing words linked to URLs. Combining the above results, citing remarks from professionals on the photo-imprinted text can be an effective strategy of persuasion, because it can make the content look more convincing. Putting the key messages in the text imprinted on photos and the source of detailed information in the post text separately can also be effective, making it easier to reach the source via hyperlinks in the post text.

Concerning public responses, engagement and comment sentiment appeared to be separate domains, revealing different responses. The photos in the “text” category ranked relatively high in mean engagement but induced the least positive comments among all categories of photos. The more words imprinted on photos, the higher the level of engagement the photos induced, but the less positive comments they had. Photo features which had an insignificant correlation with engagement showed significant associations with comment sentiment (for example, a part of face features), and the directions of correlation tended to be the opposite in the case of photo features that had a significant correlation with both engagement and comment sentiment (for example, a part of pixel and visual features). Moreover, the predictability of photo features was different in terms of engagement and comment sentiment: the former was predictable by photo features with acceptable accuracy, while the latter was not. These results suggest that messages can be designed differently depending on whether they aim to obtain more likes and comments or to induce more positive responses from the public.

Finally, some of the low-level features (pixel and visual features) showed significant correlations with public responses. The features whose high values made the colors of photos splendid were negatively associated with engagement, but those whose high values make photos look bright were positively correlated with engagement. The opposite must be noted regarding comment sentiment, as discussed in the above; the features whose high values would make the colors of photos splendid were positively associated with comment sentiment, but those whose high values made photos look bright were negatively correlated with comment sentiment. These results suggest that photos with particular low-level characteristics can appeal more to the public, and that activists ought to have this in mind when aiming to use photos as their medium.

Concerning antivaccination movements, it needs to be noted that it can be considered as disinformation based on conspiracy theories, hoaxes, and rumors. It is not only an example of confirmation bias in social media but also disorienting public opinion or generating noise. Thus, it should be warned equally against the side-effects of social media that enable connections among only like-minded people and social activities based on anti-social norms. The combination of these two threats would cause serious harm to individuals’ health as well as to society at large. In this regard, future studies will have to explore the motivation, context, and personal experiences concerning why social media users are engaged in these confirmation biases in general and the antivaccination movement in particular. Additionally, we can investigate in future studies how the word difference (e.g., scientific vs. emotional words [85,86]) in social media posts would influence the response from the public.

The major implication of this study is that it investigated how photos are used for hashtag activism. Despite their rapidly growing share in social media data and the increasing role they have been playing in online communication, visual materials have drawn relatively little attention from researchers of online activism. This study tackled the issue of using Instagram photos for hashtag activism and contributes to the body of literature by extending the research domain. Furthermore, it examined the content of antivaccination Instagram photos and revealed the characteristics of their way of delivering messages. In addition, this study showed that public responses to antivaccination Instagram photos can be manifested in two domains—engagement and comment sentiment—which can be different to each other. It can also be meaningful with regard to the photo features that were used for the analysis; low-level (pixel-level features) features as well as high-level (content-level) features can be a useful route through which implicit information can be conveyed, and analyzing them can produce other kinds of results than those generated by content analysis. A major limitation of this study is that it dealt with only one instance of hashtag activism. The approach used in this study is expected to be extended to analyze other hashtag activisms of various kinds—particularly those through photos—by comparing them with one another.

## 6. Conclusions

The aim of the present research was to analyze Instagram photos with antivaccination hashtags as an example of hashtag activism through photos and examine how the photo features were related to the public response, which was manifested via engagement and comment sentiment. This study identified that the photos categorized as “text” took the largest share. It was also found that the major way of delivering messages was to imprint the key messages that persuade people not to vaccinate with remarks from professionals on the photos and provide a source of complementary information in the post text with hashtags of antivaccine intention. Various photo features showed associations with engagement and comment sentiment, but the directions of correlation were usually the opposite: these results suggest that engagement and comment sentiment were separate domains that revealed the different public responses.

## Figures and Tables

**Table 1:** Top 30 central words of the content tag network by weighted degree centrality, betweenness centrality, closeness centrality, and eigenvector centrality.

Ranking	Weighted Degree	Betweenness	Closeness	Eigenvector
1	text	text	text	text
2	person	person	person	person
3	human face	indoor	indoor	indoor
4	screenshot	outdoor	outdoor	outdoor
5	clothing	food	clothing	clothing
6	indoor	clothing	wall	human face
7	smile	screenshot	food	wall
8	cartoon	wall	screenshot	man
9	outdoor	animal	human face	screenshot
10	man	floor	ground	woman
11	poster	ground	sky	ground
12	woman	table	man	smile
13	toddler	sky	floor	sky
14	baby	fashion accessory	woman	tree
15	book	grass	grass	grass
16	wall	human face	tree	floor
17	drawing	tree	table	book
18	design	cartoon	smile	sitting
19	abstract	man	book	cartoon
20	tree	woman	cartoon	toddler
21	grass	camera	sitting	table
22	posing	fruit	water	food
23	sky	plant	toddler	baby
24	newspaper	water	baby	building
25	sketch	cup	animal	fashion accessory
26	food	design	building	poster
27	handwriting	book	fashion accessory	girl
28	animal	sport	poster	holding
29	child	plate	holding	child
30	bottle	fast food	design	water

**Table 2:** Part of words in subgroups of the content tag network. To make it easier to see, only nodes with the top 10% of weighted degree (occupying 95.01% of the total weighted degree) were included.

Theme	Words
text and joy	text, screenshot, cartoon, poster, book, drawing, design, abstract, newspaper, sketch, food, handwriting, animal, bottle, sign, table, carnivore, illustration, child art, dog, soft drink, art, cup, graphic, drink, painting, fast food, mammal, typography, plate, cat, fruit, baked goods, coffee, map, snack, dessert, toy, monitor, billboard, music, cake, bird, letter, tableware, television, vegetable, funny, birthday cake, anime, weapon, green, juice, whiteboard, screen, black, menu, orange, concert, comic, pink, electronics, cocktail, lemon, coffee cup, shelf, tin can, mug, beer, Christmas tree
personal and indoor life	person, human face, clothing, indoor, smile, outdoor, man, woman, toddler, baby, wall, tree, grass, posing, sky, child, ground, standing, floor, fashion accessory, suit, glasses, sitting, vehicle, boy, land vehicle, car, water, group, hat, girl, building, little, face, people, footwear, furniture, bed, computer, road, holding, tie, wearing, wheel, selfie, dress, fedora, young, beach, flower, active shirt, eyes, cowboy hat, jeans, sunglasses, t-shirt, shirt, ceiling, black and white, sleeve, couch, laptop, chair, sports equipment, snow, sun hat, kiss, window, plant, vase, dance, goggles, wedding dress, cloud, jacket, swimming, trousers, smiling, presentation, sofa, laying, sport, sports uniform, bride, mountain, auto part, bicycle, helmet, swimwear, soccer, ball, field, shorts, bicycle wheel, ship, red, nature, lake, top, street, fashion, bathroom, lipstick, cellphone, football, brassiere, hand, room, athletic game, phone, scene, different, sink, sweatshirt, playground, human beard, coffee table, watch, grave, way, cemetery, transport, undergarment, bus, necktie, necklace, cosmetics
medical	medical equipment, medical, health care

**Table 3:** Correlations of photo features with engagement.

Kind	Feature	Like	Comment	Engagement
Face features	Number of faces	0.003	0.005	0.003
	Closeup	−0.005	0.011 *	−0.005
	Face ratio	−0.006	0.010 *	−0.006
	Age	0.008 *	−0.001	0.008 *
	Female	−0.001	0.014 *	0.000
	Anger	−0.001	−0.005	−0.001
	Contempt	0.004	0.004	0.004
	Disgust	0.001	0.008 *	0.002
	Fear	0.000	−0.002	0.000
	Happiness	−0.005	0.008 *	−0.005
	Sadness	−0.002	0.007 *	−0.002
	Surprise	0.002	0.002	0.002
	Neutral	0.021 *	0.010 *	0.021 *
OCR feature	Number of words	0.011 *	0.035 *	0.012 *
Pixel features	Red mean	0.024 *	0.021 *	0.025 *
	Red var	−0.004	−0.013 *	−0.004
	Green mean	0.028 *	0.023 *	0.028 *
	Green var	0.001	−0.011 *	0.000
	Blue mean	0.027 *	0.025 *	0.028 *
	Blue var	0.008 *	−0.009 *	0.008 *
	Saturation mean	−0.028 *	−0.030 *	−0.028 *
	Saturation var	−0.007 *	−0.025 *	−0.008 *
	Value mean	0.020 *	0.018 *	0.020 *
	Value var	−0.001	−0.007 *	−0.001
	Red share	−0.010 *	−0.004	−0.010 *
	Orange share	−0.021 *	−0.010 *	−0.021 *
	Yellow share	−0.014 *	−0.011 *	−0.014 *
	Green share	−0.020 *	−0.023 *	−0.021 *
	Blue share	−0.012 *	−0.008 *	−0.012 *
	Violet share	-	-	-
	Share of warm colors	−0.025 *	−0.014 *	−0.025 *
	Share of cold colors	−0.023 *	−0.021 *	−0.023 *
	Hue peaks	−0.003	−0.015 *	−0.003
	Pleasure	0.013 *	0.010 *	0.013 *
	Arousal	−0.030 *	−0.031 *	−0.031 *
	Dominance	−0.026 *	−0.025 *	−0.026 *
Visual features	Brightness	0.027 *	0.024 *	0.028 *
	Colorfulness	−0.022 *	−0.028 *	−0.022 *
	Naturalness	0.000	−0.009 *	0.000
	Contrast	0.006	−0.004	0.006
	RGB Contrast	0.001	−0.010 *	0.001
	Sharpness	−0.005	0.020 *	−0.005
	Color diversity	0.008 *	−0.021 *	0.007 *
	Color harmony	0.011 *	−0.004	0.011 *

* *p* < 0.05.

**Table 4:** Root mean square error of the 10-fold cross validation of support vector regression to engagement.

Feature	Like	Comment	Engagement
Face features	10.368	2.249	10.584
OCR feature	10.366	2.25	10.582
Pixel features	10.363	2.249	10.580
Visual features	10.355	2.248	10.572
All features	10.352	2.245	10.568

**Table 5:** Correlations of photo feature and comment sentiment.

Kind	Feature	Sentiment
Face features	Number of faces	0.020 *
	Closeup	0.002
	Face ratio	0.005
	Age	0.011 *
	Female	0.037 *
	Anger	0.004
	Contempt	−0.008
	Disgust	−0.012 *
	Fear	−0.011 *
	Happiness	0.046 *
	Sadness	−0.023 *
	Surprise	−0.016 *
	Neutral	−0.027 *
OCR feature	Number of words	−0.129 *
Pixel features	Red mean	−0.046 *
	Red var	−0.028 *
	Green mean	−0.056 *
	Green var	−0.040 *
	Blue mean	−0.066 *
	Blue var	−0.044 *
	Saturation mean	0.059 *
	Saturation var	0.006
	Value mean	−0.044 *
	Value var	−0.026 *
	Red share	0.024 *
	Orange share	0.074 *
	Yellow share	0.038 *
	Green share	0.054 *
	Blue share	−0.005
	Violet share	-
	Share of warm colors	0.081 *
	Share of cold colors	0.028 *
	Hue peaks	0.017 *
	Pleasure	−0.030 *
	Arousal	0.066 *
	Dominance	0.057 *
Visual features	Brightness	−0.056 *
	Colorfulness	0.037 *
	Naturalness	0.027 *
	Contrast	−0.046 *
	RGB Contrast	−0.040 *
	Sharpness	0.021 *
	Color diversity	0.041 *
	Color harmony	−0.011 *

* *p* < 0.05.

**Table 6:** Root mean square error of the 10-fold cross validation of support vector regression to comment sentiment.

Feature	Comment Sentiment
Face features	0.451
OCR feature	0.447
Pixel features	0.448
Visual features	0.448
All features	0.432
